# Prevalence of antibiotic resistance and integrons, *sul* and *Smqnr* genes in clinical isolates of *Stenotrophomonas* *maltophilia* from a tertiary care hospital in Southwest Iran

**DOI:** 10.22038/ijbms.2019.31291.7540

**Published:** 2019-08

**Authors:** Hadi Sedigh Ebrahim-Saraie, Hamid Heidari, Behnaz Soltani, Jalal Mardaneh, Mohammad Motamedifar

**Affiliations:** 1 Razi Clinical Research Development Center, Guilan University of Medical Sciences, Rasht, Iran; 2 Department of Microbiology, Faculty of Medicine, Shahid Sadoughi University of Medical Sciences, Yazd, Iran; 3 Department of Bacteriology and Virology, School of Medicine, Shiraz University of Medical Sciences, Shiraz, Iran; 4 Department of Microbiology, School of Medicine, Gonabad University of Medical Sciences, Gonabad, Iran; 5 Shiraz HIV/AIDS Research Center, Institute of Health, Shiraz University of Medical Sciences, Shiraz, Iran

**Keywords:** Antibiotic resistance Integrons, Smqnr gene Stenotrophomonas – maltophilia, Sul gene

## Abstract

**Objective(s):**

*Stenotrophomonas maltophilia* has emerged as an important opportunistic nosocomial pathogen due to its intrinsic and acquired resistance to a wide range of antimicrobial agents. The present study aimed to investigate the occurrence of antibiotic resistance and resistance mechanisms among clinical isolates of *S. maltophilia* from Iranian patients.

**Materials and Methods:**

This cross-sectional study was performed on 44 *S. maltophilia* isolates that were recovered from different clinical specimens in 2015 and 2016. Conventional microbiologic methods were used for primary identification of isolates and confirmed by specific polymerase chain reaction (PCR) primers. Minimum inhibitory concentrations (MICs) were determined by the E-test. PCR was applied to determine antibiotic resistance genes.

**Results:**

All of *S. maltophilia *isolates were susceptible to trimethoprim/sulfamethoxazole (TMP/SMX) and colistin. Moreover, the susceptibility rates of isolates toward ceftazidime and ciprofloxacin were 93.2%, and 84.1%, respectively. Class 1 integrons was detected in 24 (54.5%) isolates by the presence of *int1* gene. Moreover, the prevalence of antibiotic resistance genes *sul1*, *sul2*, and *Smqnr* were found in 16 (36.4%), 15 (34.1%), and 29 (65.9%) isolates, respectively.

**Conclusion:**

In summary, the prevalence of *sul* and *Smqnr* genes in integrons-contained isolates point out the significant risk of sulfonamides and fluoroquinolones resistance among clinical isolates of *S. maltophilia* in our region.

## Introduction

*Stenotrophomonas maltophilia* is an aerobic non-fermentative Gram-negative rod that is ubiquitous in natural and hospital environments (1). This bacterium has emerged as an important opportunistic nosocomial pathogen due to its intrinsic and acquired resistance to a wide range of antimicrobial agents such as β-lactam antibiotics, cephalosporins, carbapenems, and aminoglycosides (2). *S. maltophilia* can cause a wide variety of infections including respiratory tract infections (RTIs), urinary tract infections (UTIs), bloodstream infections (BSIs), skin and soft tissue infections (SSTIs), endophthalmitis, and meningitis (3). The incidence of hospital-acquired *S. maltophilia* infections is increasing and ranks third among the multidrug-resistant (MDR) non-fermentative bacteria including *Pseudomonas aeruginosa* and *Acinetobacter* *baumannii* (4). In this regard, recently in our region, the occurrence of hospital-acquired *S. maltophilia* infections has been reported (5). 

Trimethoprim/sulfamethoxazole or co-trimoxazole (TMP/SMX) is the primary drug of choice for treatment of *S. maltophilia* infections based on *in vitro* susceptibility data, and reports of favorable clinical outcomes (6). However, during recent years, there have been increasing reports of emergence of TMP–SMX resistant *S. maltophilia *(7). The reduced susceptibility of *S. maltophilia* to antibiotics is attributed to intrinsic resistance factors or the acquisition of resistance determinants through horizontal gene transfer (HGT) such as integrons and plasmids (8). The *sul* genes encoding dihydropteroate synthases are known to be responsible for resistance to TMP/SMX and have been reported to associate with class 1 integrons and insertion sequence common region (ISCR) elements (9). The *sul1* gene is predominantly linked with class 1 integrons, while *sul2* is mostly found on the plasmid (9, 10). Moreover, the *dfrA* gene which is located in the gene cassettes of the class 1 integrons has also led to high-level resistance to TMP-SXT (3). 

The fluoroquinolones are among the few antibacterial agents that showed promising activity against S*. maltophilia*; however, resistance against these antibiotics has increased in the last few years (11). *S. maltophilia* contains a novel chromosomally-encoded *S. maltophilia* *qnr* gene named *Smqnr*, which confers low-level resistance to quinolone antibiotics (12). This gene encoding protein contains pentapeptide repeats that protect DNA gyrase and topoisomerases against fluoroquinolones (12). Previously, Malekan *et al.* as the only available report on the prevalence of antibiotics resistance mechanisms among clinical isolates of *S. maltophilia* in the capital of Iran showed the presence of *sul* and *Smqnr* genes in integrons containing isolates (13).

Because of high efficiency at up-regulating or acquiring antibiotic resistance genes, Gram-negative bacteria are a particular concern for public health (14). Moreover, due to the lack of local information on the prevalence of antibiotic resistance and related mechanisms, we aimed to investigate the occurrence of antibiotic resistance and antibiotic resistance mechanisms among clinical isolates of *S. maltophilia* from hospitalized patients in Southwestern Iran. These findings can provide a good epidemiological background to contribute to the international data of *S. maltophilia* antibiotic resistance in clinical settings.

## Materials and Methods

***Research location and strategy***

This cross-sectional study was performed on a total of 44 consecutive and non-duplicated (one per patient) *S. maltophilia* isolates from hospitalized patients in Nemazee Teaching Hospital from August 2015 to March 2016. *S. maltophilia* isolates were recovered from different clinical specimens including blood, endotracheal tube, sputum, peritoneal, and nasal discharge. Nemazee is a major teaching hospital affiliated to Shiraz University of Medical Sciences located in Southwestern Iran. This study was approved by the Ethics Committee of Shiraz University of Medical Sciences (IR.SUMS.REC.1394.S573), and is in compliance with the declaration of Helsinki.

***Specimens and*** ***bacterial identification***

The suspected non-glucose fermenting Gram-negative bacilli isolates were identified as *S. maltophilia* by using the colonial morphology on blood agar (Merck, Germany), positive DNase test, and API 20E strip (API-bioMérieux, France) and confirmed by primers targeting the 23S rRNA gene as previously described (15). The identified isolates were kept at -80 °C for further investigations.

**Figure 1:**
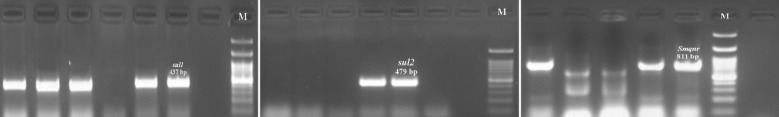
Agarose gel electrophoresis of polymerase chain reaction (PCR) products for *sul1*, *sul2*, and *Smqnr* genes. In figures, M: 100 bp DNA size marker

**Table 1:** List of used primers in the present study

**Primer**	**Oligonucleotide sequence (5′ to 3′)**	**Gene**	**Reference**
23S rRNA-F	GCTGGATTGGTTCTAGGAAAACGC	23S rRNA	(15)
23S rRNA-R	ACGCAGTCACTCCTTGCG		
IntI1-F	GGTCAAGGATCTGGATTTCG	*intI1*	(19)
IntI1-R	ACATGCGTGTAAATCATCGTC		
IntI2-F	CACGGATATGCGACAAAAAGGT	*intI2*	(19)
IntI2-R	GTAGCAAACGAGTGACGAAATG		
IntI3-F	AGTGGGTGGCGAATGAGTG	*intI3*	(19)
IntI3-R	TGTTCTTGTATCGGCAGGTG		
Sul1-F	CTTCGATGAGAGCCGGCGGC	*sul1*	(18)
Sul1-R	GCAAGGCGGAAACCCGCGCC		
Sul2-F	TCGTCAACATAACCTCGGACAG	*sul2*	(18)
Sul2-R	GTTGCGTTTGATACCGGCAC		
Smqnr-F	ACACAGAACGGCTGGACTGC	*Smqnr*	(12)
Smqnr-R	TTCAACGACGTGGAGCTGT		

**Table 2:** Demographic data of studied patients with *Stenotrophomonas maltophilia* infection

**Characteristic**		No.	%
**Age **	Mean ± SD	25.3 ± 32.1 years	
	Median	6 years	
	Range	1 month to 90 years	
**Gender**	Male	24	54.5
	Female	20	45.5
**Isolation ward**	NICU	23	52.3
	ICU	14	31.8
	General medicine	5	11.4
	Transplant	1	2.3
	Surgery	1	2.3
**Source of infection**	BSI	39	88.6
	RTI	5	11.4

**Abbreviations:** BSI: bloodstream infection, RTI: respiratory tract infection; NICU: neonatal intensive care unit; ICU: intensive care unit

**Table 3:** Minimum inhibitory concentrations (MICs) and susceptibility profiles of *Stenotrophomonas maltophilia* for the tested antimicrobial agents

**Antibiotic**	MIC in µg/mL															
	0.094	0.125	0.19	0.25	0.38	0.5	0.75	1	1.5	2	3	4	6	8	12	≥32
TMP/SMX	2	11	21	5	**4**	1										
Ciprofloxacin	2	5	2	11	7	2	4	4	1	**3**	3					
Ceftazidime						1	1	4	4	6	11	7	4	**3**	2	1
Colistin			9	19	9			1	**2**	4						

MIC50s are underlined, and MIC90s are in boldface

**Abbreviations: **TMP/SMX: trimethoprim/sulfamethoxazole

**Table 4:** The distribution of antibiotic resistance genes based on the source of isolation

**Source of infection**	**Class 1 integrons** **No. (%)**	***sul1*** **No. (%)**	***sul2*** **No. (%)**	***Smqnr*** **No. (%)**
BSI (N = 39)	21 (53.8)	14 (35.9)	14 (35.9)	25 (64.1)
RTI (N = 5)	3 (60)	2 (40)	1 (20)	4 (80)
Total (N = 44)	24 (54.5)	16 (36.4)	15 (34.1)	29 (65.9)

**Abbreviations:** BSI: bloodstream infection, RTI: respiratory tract infection

***Antimicrobial susceptibility testing***

Minimum inhibitory concentrations (MICs) were determined by the E-test (Lioflichem, Italy) as described by the Clinical and Laboratory Standards Institute’s (CLSI) recommendation (16). The following agents were included: trimethoprim/sulfamethoxazole (TMP/SMX), ciprofloxacin, ceftazidime, and colistin. CLSI 2017 interpretive criteria were used for each antibacterial agent. Control strains for susceptibility testing were *Pseudomonas aeruginosa* ATCC 27853. Ciprofloxacin and colistin breakpoints of CLSI 2017 for *Acinetobacter* spp. were used to determine susceptibility.

***DNA extraction and Molecular genotyping***

The boiling method was used to extract genomic DNA as described previously (17). Simplex polymerase chain reaction (PCR) was used to determine the presence of sulfonamide (*sul1* and *sul2* genes) and quinolones (*Smqnr*) resistance genes and class 1 to 3 integrons by specific primers for integrase genes (*intI1*, *intI2*, and *intI3*) as described previously (12, 18, 19). All used primers in the present study are listed in Table 1; they were all obtained from Pishgam Biotech Co, Tehran, Iran. The cycling conditions were set up as follows: 5 min at 95 °C as initial denaturation, 30 cycles of denaturation at 95 °C for 60 sec, annealing (the temperature depended on the primer sequence), extension at 72 °C for 50 sec, and final extension at 72 °C for 5 min on a T100™ thermal cycler (Bio-Rad, Hercules, CA, USA). PCR products were separated by electrophoresis in 1.5% agarose gels with 1X TAE (Tris/Acetate/EDTA) buffer. DNA bands were observed by staining with safe stain load dye (CinnaGen Co., Iran) and visualized under UV illumination. The purified PCR products for each gene were submitted for sequencing (Bioneer Co., South Korea), and sequences were compared using online BLAST software (http://www.ncbi.nlm. nih.gov/BLAST/).

***Statistical analyses***

Statistical analysis was performed using SPSS^TM^ software, version 21.0 (IBM Corp., USA). The results were presented as descriptive statistics in terms of relative frequency. Chi-square or Fisher’s exact tests were used to analyze the results wherever needed. *P*-value <0.05 was considered statistically significant.

## Results

Totally, 44 clinical isolates of *S. maltophilia* were included in the present study. *S. maltophilia* isolates were obtained from 24 (54.5%) male and 20 (45.5%) female subjects with a mean age of 25.3 ± 32.1 years, ranging from 1 month to 93 years. The most frequent source of bacterial isolation was from BSIs 33 (88.6%) followed by RTIs 5 (11.4%). The majority of patients were hospitalized in neonatal intensive care units (NICUs) (52.3%) followed by intensive care units (ICUs) (31.8%), and general medicine wards (11.4%). Demographic data of patients is summarized in Table 2.

The full results of antibiotic susceptibility testing including MIC50 and MIC90 (MIC at which 50% and 90% of isolates were inhibited) of the tested isolates are shown in Table 3. Overall, all isolates of *S. maltophilia* were susceptible to TMP/SMX and colistin. Moreover, the susceptibility rates of isolates toward ceftazidime and ciprofloxacin were 93.2% and 84.1%, respectively. The MIC50/MIC90 of TMP/SMX, ciprofloxacin, ceftazidime, and colistin were 0.19/0.38 µg/ml, 0.38/2 µg/ml, 3/8 µg/ml, and 0.25/1.5 µg/ml, respectively. 

PCR amplification of the three classes of integrons genes showed that 24 (54.5%) of isolates carried class 1 integrons by detection of the *intI1* gene. Neither class 2 nor class 3 integrons were found among all of the isolates by the absence of *intI2* and *intI3 *genes. Sulfonamide resistance determinants, *sul1* and *sul2,* were detected in 16 (36.4%) and 15 (34.1%) of isolates, respectively. Meanwhile, the co-occurrence of *sul1* and *sul2* genes was detected in nine (20.5%) isolates, and totally 22 (50%) isolates were positive for *sul* genes. The presence of *sul1* was significantly associated with class 1 integrons (*P<*0.007), while three class 1 integrons-negative isolates carried the *sul1* gene. Finally, quinolones resistance gene (*Smqnr*) was found in 29 (65.9%) of isolates. All of the ciprofloxacin non-susceptible isolates contained the *Smqnr* gene (*P<*0.038). The distribution of antibiotic resistance genes based on the source of isolation is demonstrated in Table 4.

## Discussion

In recent years, there has been a significant increase in the occurrence of* S. maltophilia* nosocomial infections, particularly in ICUs (5, 6, 20). This unfortunate trend is a consequence of underlying debilitating diseases and poor immunity state of ICU patients and other factors including extensive use of antibiotics (5, 6, 21).

Due to the intrinsic antibiotic-resistant nature of *S. maltophilia*, the therapeutic options are limited to a few antibiotic agents including TMP/SMX, ceftazidime, fluoroquinolones, ticarcillin-clavulanate, and minocycline (22). In our results, fortunately, the rates of resistance toward the drug of choice for treatment of *S. maltophilia* infections were scarce. Previously, three separate reports from north of Iran showed that the susceptibility rate of clinical isolates toward TMP/SMX was 82–100%, 80–100% for fluoroquinolones, while this rate for ceftazidime was 0–82% (13, 23, 24). Despite reports comparable to our findings, antibiotic resistance rates vary geographically. In this regard, our results are closest to the median values of susceptibility rates reported in several Asian countries including China, Taiwan, Thailand, Malaysia, Korea, and Japan, where susceptibility to TMP/SMX was 66.7–98.4%, 67.2–79.6% for fluoroquinolones, and 24.4–62.5% for ceftazidime (6, 25-29). However, compared to results reported by Farrell *et al.* from a worldwide collection of *S. maltophilia*, it seems that antibiotic resistance rates in Europe and American regions are slightly lower than in Asian countries (30). Moreover, in agreement with our findings, several studies showed promising *in vitro* activity of colistin against clinical isolates of *S. maltophilia *(31, 32); however, increase in trend of colistin-resistant strains was also noted in some region (33).

As part of the HGT system, integrons have a significant role in the spread of resistance genes among the bacterial population (34). In our results, the prevalence of class 1 integron-integrase gene was estimated at 54.5%. The global prevalence of integrons-positive isolates of *S. maltophilia *can be found in the literature from different sources; however, the reported rates showed a great variation mostly linked to geographical distribution or source of isolation. Malekan *et al.* from North of Iran showed the prevalence of class 1 integrons in 14% of *S. maltophilia *isolates that were resistant to TMP/SMX (13). The prevalence of class 1 integrons in our study is lower than those reported in Korea (6%) (29), India, (8.5%) (35), Turkey (12%) (36), Egypt (40.6%) (37), and Japan (45.5%) (38), whereas it is lower than those reported by two separate studies in China (64.7%, and 72.7%) (26, 39). 

Two allelic forms of sulfonamides resistance determinants, *sul1* and *sul2* genes, have been associated with TMP-SMX resistance in *S. maltophilia *(9); therefore, the information regarding their distribution is essential to gain a close estimation of the burden of sulfonamide-resistant strains for the development of effective healthcare practice. There is a significant variation in the reported prevalence of the *sul1* gene; however, it seems that based on previous reports, *sul1* is mostly associated with the presence of class 1 integrons and has a higher prevalence than the *sul2* gene in clinical isolates of *S. maltophilia* regardless of TMP/SMX resistance (13, 25, 26, 29, 39). In the present study, we report the prevalence of *sul1 *and* sul2 *genes; however, none of our isolates showed *in vitro* resistance to TMP/SMX. Although, these findings are not uncommon since previously several reports showed the prevalence of *sul* genes in TMP/SMX-susceptible isolates (37, 39).

Fluoroquinolones are some of the most effective drugs used against *S. maltophilia *(1, 8). However, recent studies have demonstrated a trend in increasing resistance to fluoroquinolones (27). Previously, Malekan *et al.* in the North of Iran reported 10% of the resistant-isolates were positive for the fluoroquinolones resistance-associated gene (*Smqnr*) (13). In Asian countries, the prevalence of *Smqnr* was reported ranging from 25% in China to 57.5% in Japan (4, 27, 40). 

## Conclusion

In summary, the prevalence of *sul* and *Smqnr* genes in integrons-contained isolates points out the significant risk of sulfonamides and fluoroquinolones resistance among clinical isolates of *S. maltophilia* in our region. However, future investigation to find every other possible mechanism of resistance is necessary before reaching any comprehensive conclusion.
